# Educational improvement through machine learning: Strategic models for better PISA scores

**DOI:** 10.1371/journal.pone.0326121

**Published:** 2025-07-02

**Authors:** Bilal Baris Alkan, Serafettin Kuzucuk, Şevki Yetkin Odabasi, Leyla Karakuş

**Affiliations:** 1 Department of Educational sciences, Akdeniz University, Antalya, Turkey; 2 Antalya Measurement and Evaluation Center Directorate, Antalya, Turkey; Parantez Education Research Consultancy and Publishing Services, TÜRKIYE

## Abstract

In this study, in addition to traditional variables such as economic wealth or the number of books read, on which many studies have already been conducted, variables that are thought to influence student achievement and better predict success are identified. Random Forest algorithm was used to identify important variables based on the PISA 2018 data, covering all three domains of science, mathematics and reading. The study found that the main factors influencing the success of students in countries that perform well in the PISA exam are essentially access to information technology, weekly hours of instruction in the subject, economic-social and cultural status, parents’ occupation, level of metacognition, awareness of PISA, sense of competition and attitudes towards reading. New prediction models based on these variables were proposed. The proposed models will give a significant advantage to policy makers who want to improve their country’s PISA score and implement appropriate education policies.

## Introduction

Today, various measurement and evaluation tools are used worldwide to analyze and compare both the quality of educational practices and student achievement [1] The best known and most effective comparative assessment program among them is the Program for International Student Assessment (PISA) [2]. OECD countries benefit greatly from the results of this program when evaluating and improving their education systems. PISA, which is conducted every three years by the OECD, assesses the skills of 15-year-old students in reading, mathematics and science and provides a valuable basis for international comparisons [3]. In contrast to traditional measurement and assessment methods, PISA focuses on assessing students’ critical thinking and practical problem-solving skills. In this sense, it aims to measure not only students’ access to knowledge, but also how they process and apply it in real-world contexts. As the need to raise educational standards in a globalized world becomes ever greater, PISA has grown in importance [4]. The results of the program provide an objective basis for understanding the position and performance of a country’s education system in an international comparison and promote a climate of competition [5]. In addition, this data allows countries to assess structural differences, policy orientations and implementation outcomes in their education systems. PISA also facilitates the investigation of the causes of individual success or failure and provides important insights for the development of strategies to improve student performance [6]. In order to gain relevant insights, the PISA data has been used in a variety of publications/studies for various analyzes and comparisons. A close examination of the purpose and content of these studies reveals that most focus on comparing countries’ performance in science, reading and maths and attempt to identify the determinants of success or failure in order to inform educational policy makers [7–12]. Studies that aim to improve the quality of learning processes and provide meaningful insights have also gained attention [10,13–15]. However, much of this literature relies on a limited number of variables and rarely applies advanced or innovative analytical techniques, so it has limited explanatory power. In general, student achievement is explained by commonly accepted variables such as socioeconomic status or the number of books in the household, which are well known and often do not require robust empirical testing. This underscores the need for researchers and policy makers to explore additional factors- that are as important or even more effective than socioeconomic status and that contribute to student achievement.

In this context, the rich, multivariate and culturally diverse structure of the PISA data requires not only traditional statistical analysis, but also the application of artificial intelligence-based algorithms. While many studies limit themselves to regression or correlation analyzes [16,17], they often overlook non-linear relationships, interactions between variables and contextual dependencies. In recent years, the increasing use of machine learning algorithms in educational research has shown that they have great potential to explain complex phenomena and make accurate predictions [18,19].

On the other hand, recent studies have shown that methods such as the Random Forest Algorithm (RFA) offer unique advantages in this area. In particular, its ability to detect nonlinear interactions and assess the relative importance of variables in large training datasets is remarkable [20–22]. While traditional approaches often ignore complex dependencies and interaction effects, RFA can uncover complex patterns that classical statistical models cannot detect. This property has made it a particularly valuable tool for comparative educational research. However, despite all this potential, the use of RFA in PISA-based studies is still limited, pointing to an important methodological gap that our study attempts to fill [23].

The main objective of this study is to identify the factors that influence student achievement in OECD countries with above-average performance in reading, maths and science, beyond traditional variables such as economic wealth or the number of books read, and to develop a predictive model based on these variables that can provide functional insights to policy makers in countries seeking to improve their PISA results. The following research questions guide the study:

RQ1) What are the most important variables influencing OECD countries’ PISA results?

RQ2) Can a predictive model based on these critical variables serve as an early warning system for countries seeking to improve their PISA performance?

With this in mind, to answer the relevant research questions in the study, an application of the Random Forest Algorithm was conducted with 24 categorical variables based on PISA (2018) results in science, reading and mathematics and student information for 37 OECD countries. RFA as an ensemble learning method consisting of multiple decision trees has been shown to be effective in both classification tasks and in determining the significance of variables [24]. Especially in complex social systems such as education, where traditional statistical models may not be able to capture nonlinear and high-dimensional relationships, RFA offers significant advantages. Its ability to work with large data sets, tolerate missing values and deal with multicollinearity has led to its increasing use in the field of educational data mining [25]. In this study, RFA was used not only to create powerful predictive models, but also to provide decision makers with evidence-based prioritization of influential variables. As a result of the analysis conducted using the R software, six variables for reading, six for mathematics and eight for science were identified as the most important factors influencing student performance. These variables included access to information technology, weekly hours of instruction in the respective subject area. Economic-social and cultural status, parents’ occupation and level of metacognition were found to be important and common variables for all three areas. Other variables that have an independent effect only on the respective domain are weekly hours of instruction in a foreign language for the reading domain; a sense of competition for the math domain; for the science domain, awareness of PISA was found to be the perception of difficulty and the emotional state related to the act of reading. Consequently, the determination of these variables is very important, especially when it comes to helping teachers and education authorities in the countries concerned to make correct and effective decisions in policy-making processes. In this context, it is clear that the study has a supportive and guiding character.

To support this aim and ensure a coherent flow of research, the structure of the article has been designed to reflect a logical sequence from context to conclusion. The Literature Review section discusses the contribution of PISA data to educational policy, identifies the key variables influencing student achievement and explores the limitations of traditional methods of analysis, providing a sound theoretical and empirical basis for the study. In this context, the importance of machine learning-based approaches --in particular the Random Forest Algorithm (RFA)-- is emphasized and the reasons for its selection are explained. The “Materials and methods” section provides a detailed description of the PISA 2018 dataset used in the study, covering aspects such as the selection of the sample, the coding of the variables and the data pre-processing procedures. It also outlines the reasons for the choice of the RFA and explains how it was implemented in the analysis. The “Results and findings” section presents the variables that have the greatest impact on student achievement in reading, math, and science. The relative importance of these variables and their common or domain-specific effects are interpreted and illustrated using visual representations. Finally, the “discussion and conclusions” section situates the findings within the wider literature and evaluates the methodological contributions of the study. Practical recommendations for policy makers are proposed, and directions for future research are suggested.

## Background on PISA

The effects of recent technological developments can be clearly felt in educational research. Especially with the help of current technological methods and techniques such as data mining and machine learning, there are a large number of studies conducted with the aim of evaluating the quality of the education system, accelerating the learning and teaching process, investigating the factors that influence the quality of learning activity or directly affect learning success, and contributing to policy development processes in this direction. Among these studies, those based on the PISA data are particularly noteworthy because their scope and impact are international. Table 1 lists the studies that have been conducted based on PISA data to identify the factors that affect student’ learning outcomes and to suggest how education policies should be designed in this regard.

**Table 1:** PISA-based academic studies.

Reference	Year	Purpose
Fredriksson et al.	2009	Compares the strengths and weaknesses of the Swedish and Swiss education systems. It provides information on an international level by examining the education policies of different countries.
Cosgrove and Cartwright	2014	Examines the changes in PISA results in Ireland. It is an important study to understand how international evaluations can influence education policy at country level.
Waldow et al.	2014	Examines how media discourses based on PISA results influence education policy in Germany, Australia and South Korea. Examines how perceptions of educational performance are shaped by countries labeled as “Asian Tigers”.
Jiang and McComas	2015	It assesses the impact of research questions on student’ science achievement and attitudes. It uses PISA data to understand the impact of teaching strategies on students.
Schatz et al.	2015	It examines the Finnish education system as it moves from PISA to national branding. It assesses how the Finnish education system is perceived and how it shapes the country’s brand.
French et al.	2015	This study, which examines the relationship between cultural dimensions, education expenditure and PISA performance, provides an important analysis for understanding the role of cultural factors in education.
Agasisti and Longobardi	2016	It is an empirical study of educational equity, school characteristics and student resilience in the EU-15 countries. Based on the OECD’s 2009 PISA data, it provides important information for education policy by examining the factors that influence educational inequalities and student achievement.
Baroutsis and Lingard	2016	Analyzes how the PISA results are presented in the Australian media. Assesses the influence of the media on educational performance and identifies policy decisions that may influence public opinion.
Morgan and Volante	2016	Examines OECD international education research, focusing on discourses on human capital, governance and policy debates. Evaluates and compares international education policy.
Ringarp	2016	Examines how PISA has created legitimacy and influenced changes in education policy in Germany and Sweden. Evaluates how PISA contributes to changes in education policy.
Schleicher and Zoido	2016	Examines the policies that shape and are shaped by PISA. Assesses how international education policy can be shaped by examining how PISA is used and how it influences education policy.
Niemann et al.	2017	Examines how PISA can influence education policy. Provides policy recommendations by comparing education systems at the international level.
Sälzer and Roczen	2018	Examines the methods used to assess global competence in PISA 2018. Assesses how we can tackle complex structures and make sense of the PISA data.
Eklund et al.	2018	PISA examines the early cognitive factors that influence reading achievement, with a particular focus on children at family risk.
Tonga et al.	2019	It looks at the professional development of teachers in the countries participating in PISA. It assesses the professional development of teachers using data from Finland, Estonia, Japan, Singapore and China.
Fitzpatrick et al.	2019	This research on answer sets, exemplars and authenticity that do not help children solve real-world problems provides a valuable insight into understanding learning processes.
Sánchez et al.	2019	This study, which analyzes the moderating effect of gender, examines the explanatory factors that predict academic success on the PISA tests.
Auld et al.	2020	Analyzes the results of the pilot implementation of PISA. The study examines how PISA data can be used to understand changes in education and make recommendations for education policy.
Entrich	2020	It explains differences in socio-economic inequalities by examining shadow education practices around the world. It provides a valuable analysis for understanding the relationship between shadow education and social inequality.
Lopez and Gamazo	2020	It analyzes the 2015 Mexican PISA results and assesses the variables that explain student performance. It is an important resource for understanding education policy in Mexico.
Odell et al.	2020	It assesses the impact of technology in education by examining the relationship between students’ information technologies and their performance in math and science.
Ding and Homer	2020	This study, which interprets math performance in PISA by taking into account reading performance, is an important contribution to a more comprehensive understanding of student performance.
Martínez‐Abad et al.	2020	This study, which identifies factors associated with school effectiveness in PISA by analyzing educational data, helps to identify effective factors in education.
Pedreroand Manzi	2020	This study, which universally assesses subjective beliefs, connectedness, and motivation in science and math, provides an important analysis to understand the factors that influence student success.
Agasisti et al.	2021	He examined school factors associated with the success of socio-economically disadvantaged students using PISA data. This analysis sheds light on measures that can be taken at school level to understand and reduce social inequalities in education.
D’Agostino et al.	2021	It examines the relationship between anxiety and academic achievement and looks at the psychological factors that influence student success. Using PISA data, it contributes to the understanding of the role of student mental health in education.
Eriksson et al.	2021	Examines the impact of socioeconomic status on student achievement. Emphasizes the role of socioeconomic factors in understanding the academic performance of students in different societies.
Mazurek et al.	2021	Examines the relationship between inequality and student performance in PISA 2018. Evaluates inequalities between countries and the impact of these inequalities on student achievement.
Santos et al.	2021	Examines the factors that influence student success using data from PISA 2015. Analyzes the factors that lead students to success.
Delprato and Antequera	2021	Examines the effectiveness of schools in low- and middle-income countries by analyzing data from the PISA study on developmental learning.
Eryilmaz and Sandoval-Hernández	2021	This study, which examines the relationship between cultural capital and students’ perceptions of feedback using PISA 2018 data from 75 countries, makes a valuable contribution to understanding the role of cultural factors in education.
Gjicali and Lipnevich	2021	This study, which examines the (indirect) effects of student’ mathematics attitudes on intentions, behavioral engagement, and mathematics performance on the PISA exam in the United States, makes an important contribution to understanding the impact of student attitudes on learning processes.
Haw et al.	2021	In examining the lessons that the Philippines can learn from PISA 2018, this study finds that needs-based instruction is associated with better reading achievement.
Ma et al.	2021	By examining the multilevel relationship between perceived teacher support, student cognitive ability, enjoyment of reading, and reading achievement, this study provides a model for understanding the factors that influence student motivation and achievement.
Rolfe	2021	This study on the adaptation of a measurement model for socioeconomic status provides an important methodological contribution using 15 years of PISA data.
Yu and Zhao	2021	This study, which examines the negative effects of bullying victimization on academic literacy and social integration using PISA data in 51 countries, assesses the impact of bullying on student achievement and social cohesion.
Zhang	2021	This study, which examines the impact of academic social capital on student performance in science, uses PISA data from 2015 to show that social capital is an important factor in science.
Fernández-Alonso et al.	2022	In his meta-analysis study, he examines the impact of homework support for students. He assesses the role of homework on student achievement and emphasizes the importance of parental support for students.
Perry et al.	2022	Evaluates the impact of school socioeconomic status on student achievement using quantile regression analysis. Emphasizes the role of social inequalities in education.
Pitsia	2022	Focuses on the study of high achievement in mathematics and science among students in post-primary education in Ireland. Evaluates the factors influencing student achievement using binary logistic regression analysis at multiple levels.
Bhutoria and Aljabri	2022	It utilizes data from the 2018 PISA study by examining the cognitive benefits of ICT use among 15-year-old students at a global level.
Ferrera et al.	2022	Based on the 2015 PISA study conducted with the participation of OECD countries, this study provides important information on the efficiency of education systems to assess the effectiveness of secondary schools.
Jerrim et al.	2022	Against the background of new findings from a long-term PISA study conducted in England, this study examines the relationship between inquiry-based teaching and student achievement and focuses on assessing the impact of teaching methods.
Kong et al.	2022	This study, which compares the impact of information and communication technology on digital reading achievement using PISA 2018 data, provides a cross-national comparison.
Lee	2022	This study evaluates the performance of China’s urban and rural secondary schools and provides important insights by applying a machine learning approach using PISA 2018 data.
Münch and Wieczorek	2022	In its pursuit of quality and equity, this study provides important perspectives by comparing the UK and Germany in the battle for PISA results.
Pulkkinen and Rautopuro	2022	Conducted in Finland, this study assesses the impact of the Finnish education system by examining the correspondence between PISA results and school performance.
Yang and Lee	2022	This study, which examines whether school resources reduce the socio-economic achievement gap, attempts to answer an important question using data from PISA 2015.
Hu and Wang	2022	The aim of the study is to investigate the influence of students’ perceived teaching quality on digital reading performance in 29 OECD countries. The study presents important findings in this area by assessing the relationship between student’ perceptions and performance in digital reading using a multilevel analysis.
Bayirli et al.	2023	It analyzes the mathematics performance of students in Asia-Pacific countries and shows how data mining can be used in education. Such analyzes are important for understanding student performance and planning future educational strategies.
Dai et al.	2023	PISA identifies factors that predict the reading performance of multilingual students. This results in factors that need to be considered in the education of students with different language skills.
Kim and Kim	2023	Examines the profiles of information and communication technology (ICT) use by students in high-performing countries. Using PISA data, it provides an important analysis to understand the role of technology in education.
Liu et al.	2023	It assesses the impact of learning time allocated to students on the academic performance of secondary school students. Using data from PISA 2018, it examines the time management factors that influence student success.
Santos and Centeno	2023	Examines the importance of looking abroad for inspiration by exploring how PISA influences countries selection of countries as reference societies in education. Evaluates the role of different countries in shaping their education policies.
Sommet et al.	2023	Addresses the relationship between income inequality, competitiveness and cooperation. Evaluates the role of income inequality in education and how it affects competitiveness and cooperation.
Wang et al.	2023	Provides a comprehensive analysis by systematically examining the factors that influence performance in mathematics in PISA. It assesses the impact of these factors on student performance in mathematics.
Agasisti et al.	2023	It aims to shed light on the efficiency of the education system by analyzing the use of technological resources and ICT in Latin American schools using OECD PISA 2018 data.
Dang et al.	2023	This study, which examines the reasons for Vietnam’s outstanding performance in education compared to other countries, provides important insights by analyzing PISA data from 2012, 2015 and 2018.
Steinmayr et al.	2023	By examining the long-term relationships between cognitive needs, achievement goals and school grades, this study tests a meditation hypothesis.
Vasalampi et al.	2023	By investigating whether PISA reading performance, reading motivation, and academic burnout predict adolescents’ educational trajectories and achievement, this study provides an important framework for understanding student motivation.
Wang and Wang	2023	This study, which examines the relationship between information and communication technology resources in the classroom, student engagement, and academic achievement through multilevel structural equation analysis using PISA 2018 data, is an important research contribution to understanding the role of technology in education.

If we examine the studies by purpose and year, we find that most of the studies published in the 2010s were conducted with the aim of demonstrating the contributions of PISA so that they can be used as an international basis and target for guiding education policy. It becomes clear that the benefits of the study, such as the improvement of the country’s education policies and activities and the possibility of international comparison, were taken for granted and the studies were conducted with the aim of making direct functional use of the PISA data and investigating the factors that influence student performance.

When the existing literature is examined in terms of the methodology employed, it was observed that previous studies applying the Random Forest Algorithm (RFA) to PISA data [12,20–22] generally focused on a single domain or were conducted using a limited set of variables. In contrast, the present study offered a notable methodological and practical contribution by simultaneously modelling student performance in three domains (reading, mathematics, and science), utilizing a broader variable set, and presenting the variable importance results in a comparative format. The choice of the Random Forest algorithm was not only aligned with the scope and structure of the study but also supported by its strong presence in the literature regarding its high accuracy and compatibility with categorical data. The Random Forest Algorithm (RFA) has outperformed logistic regression in analyzes with categorical data [26]. In a comparative study with 115 binary data sets and 14 different algorithms, it achieved the highest accuracy of all methods tested [27]. It also provided the most successful results compared to CART, C5.0, Naïve Bayes, Linear Discriminant Analysis and K-Nearest Neighbour in similar comparative analyzes [28]. In an analysis with PISA 2018 data, it provided higher accuracy than Hierarchical Linear Modeling [29]. It even outperformed Artificial Neural Networks and Support Vector Machines in predicting student satisfaction [30]. A review of 72 education-related studies conducted between 2015 and 2023 emphasized the strong correspondence with educational datasets and the ability to provide interpretable and meaningful predictions [11]. In light of these findings, the RFA was employed in the present study as a functional tool for identifying variable importance, constructing predictive models, and generating threshold values. Its methodological flexibility and analytical precision played a key role in achieving the core objectives of the study.

## Materials and methods

In this section of the study, which was carried out to identify the main variables affecting the successful performance of countries with a score above the OECD average in reading, mathematics, and science, and to create a prediction model for other countries aiming to increase their PISA achievement through these variables, detailed information on the research data and methodological procedure is provided.

### Obtaining data

The dataset of the study consists of PISA (2018) scores of 37 OECD countries in science, reading and mathematics and other variables including information about PISA participating students. When the data set was examined, it was seen that there was no missing data. .Subsequently, the non-categorical variables that provide information about the students were categorized. In Table 2, abbreviations and explanations related to the 24 categorical variables obtained within the scope of the PISA exam and to be used within the scope of the study are given.

**Table 2:** Information on the variables whose effect on PISA scores.

Abbreviation	Description
**HISEI**	The index value of the parent with the highest occupational status in the family.
**ESCS**	A composite indicator that reflects the student’s economic, social and cultural status.
**WEALTH**	An index composed of objects that reflect the level of material well-being of the family (car, room, dishwasher, etc.).
**CULTPOSS**	Availability of materials with cultural content (classic books, works of art, etc.) at home.
**HEDRES**	Access to educational resources at home (books, desk, internet access, etc.).
**HOMEPOS**	An index based on the presence of items related to the general standard of living and learning environment at home.
**JOYREAD**	The student’s level of enjoyment of reading and positive attitude towards reading activities.
**ADAPTIVITY**	The student’s perception of the extent to which his/her teacher adapts instruction to his/her needs.
**COMPETE**	The extent to which the student believes he is competing academically with his classmates.
**MASTGOAL**	Whether the student’s learning goals are focused on understanding and developing skills.
**METASPAM**	The student’s ability to judge the reliability of a piece of information or a source.
**PISADIFF**	The student’s personal assessment of how difficult he/she finds the PISA test.
**SCREADCOMP**	The student’s level of self-efficacy in relation to his/her own reading skills.
**SCREADDIFF**	The extent to which the student finds the reading activity challenging.
**UNDREM**	The student’s ability to understand and recall information when reading a text.
**METASUM**	The student's ability to summarise a text, extract the main idea and reformulate it.
**STIMREAD**	The student's perception of whether the teacher's behaviour encourages the student to read.
**CPERLAN**	The number of weekly lessons the student receives in the test language.
**CPERMATH**	The number of maths lessons per week.
**CPERSCİ**	The number of science lessons per week.
**CPERWEEK**	The total weekly teaching load of the pupil (in lessons).
**CPERMİN**	The average duration of a lesson (in minutes).
**CPERFORLAN**	The student's weekly foreign language lessons.
**ICTRES**	Student's access to digital technologies at home and school.

### Preparing data for analysis

The data set was subjected to a comprehensive pre-processing process before being analysed. Firstly, the continuous variables were converted into meaningful categories and made usable for the analysis. Distribution characteristics and literature applications were taken into account when categorising the variables. The variables that were considered necessary were coded and insignificant variables were removed. In addition, the names of the variables were standardised according to the abbreviations. Countries’ scores in science, reading and maths were assessed on the basis of the OECD average. 487 points in reading, 489 points in maths and 489 points in science were accepted as the OECD average. Countries that scored above these thresholds were categorised as high-performing countries, while countries that scored below were categorised as low-performing countries. Thus, the performance levels of the countries were categorised into two categories and a suitable structure for the classification algorithms was created.

### Random forest application

When analysing the data, the random forest algorithm was preferred in order to determine the significance of the variables and create a prediction model. This algorithm is a machine learning method that works on the basis of decision trees and shows high performance in both classification and regression problems [24]. Random Forest can harmonise with high-imensional data structures, minimize multicollinearity problems and objectively represent the ranking of variable importance [25].

The structure of the categorical data used in the study further emphasizes the advantages of the random forest algorithm. The algorithm in question was favoured not only in terms of predictive success, but also in terms of its ability to define complex interactions between variables. In this context, recent studies such as [20,22,23] emphasise that the RFA method is increasingly used in educational data mining.

In the analysis, the data set was transferred to the programme R (version 4.3) and the Random Forest algorithm was applied via the Boruta package. The Boruta is a wrapping method based on Random Forest and calculates the significance levels of the variables using Z-scores [31]. Thanks to this package, the predictive power of all variables in the data is tested and only the significant variables are included in the model. During the analysis, a “shadow copy” was created for each variable and its Z-scores were compared; only variables with unique information value were included in the model. This increased the accuracy of the model and the degree of explainability.

### Visualization of analysis results

**Fig 1:**
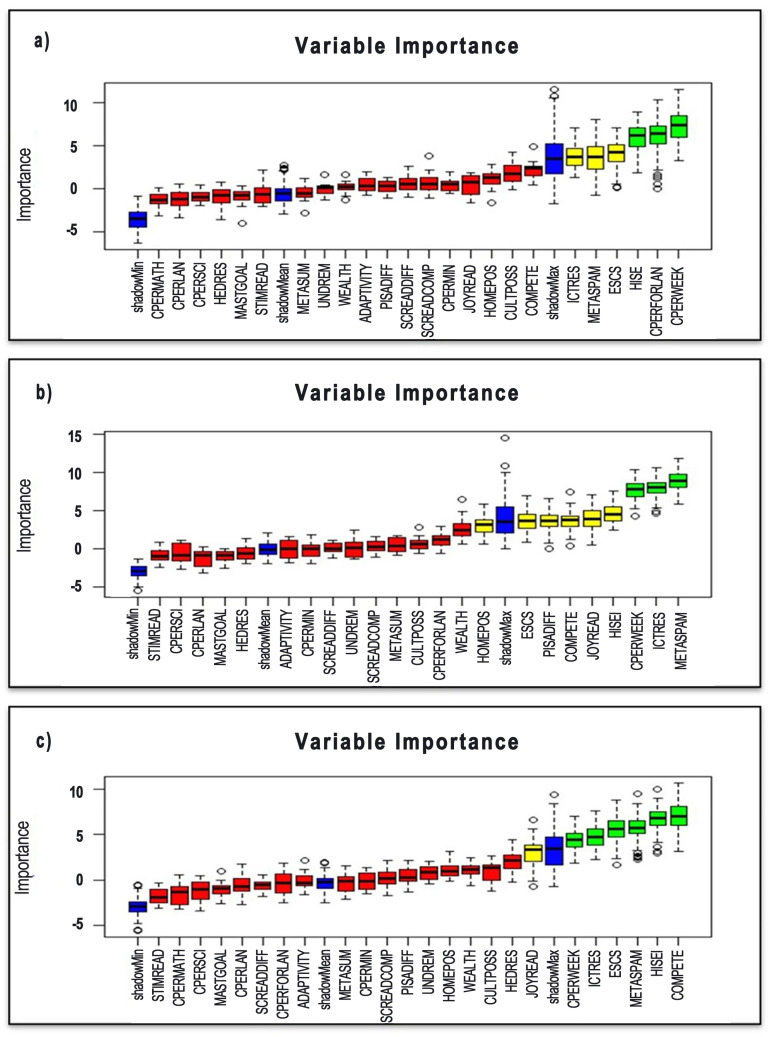
Most Important Variables in Reading(a), Science(b) and Math(c) Section.

**Fig 2:**
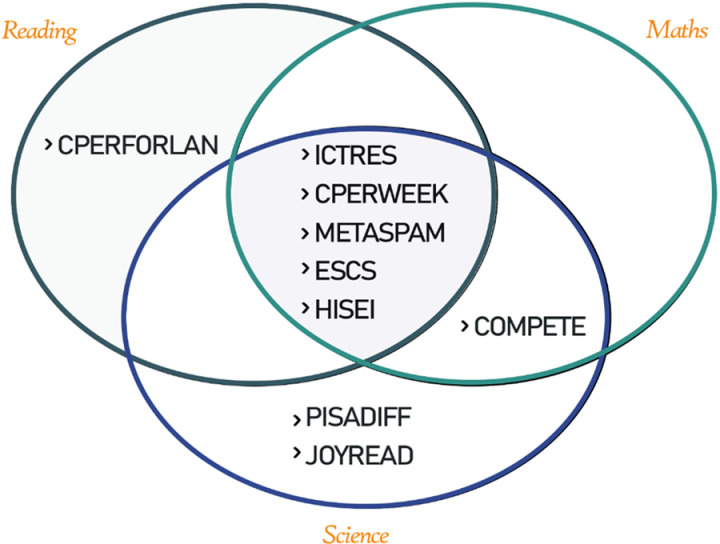
Measurement areas and important variables in PISA 2018.

In order to better understand the results of the analysis, the importance levels of the variables and their classification successes are shown graphically in Figs 1 and 2. The prominent variables for each domain (reading, mathematics and science) are visualized separately; this facilitates the comparison of the determining factors in different domains. These visualizations support both scientific interpretation and a more effective presentation of conclusions to policy makers.

## Finding and results

In this part of the study, findings and results of the data analyses conducted to answer the research questions are presented.

### RQ1: The most important variables affecting students’ PISA success

In line with the first research objective of determining the most important variables affecting students’ PISA achievement; standardised PISA scores (science, mathematics, reading) were obtained for OECD countries and a classification variable was created according to the success or failure of the countries. After the class variable was determined, the first findings regarding the results of the analysis carried out to determine which of the variables categorised in the previous stage, which were thought to have an effect on the achievement of the countries, had the most significant effect on achievement are presented in Fig 1 (a–c).

When the box plot, in which green, yellow and red coloured boxes represent important, temporarily important and unimportant variables respectively, is examined in detail, it is seen that 9 variables are important in total. ICTRES (ICT resources), CPERFORLAN (The typically required to attend: number of [class periods] per week in foreign language), CPERWEEK ( in a normal, full week at school, how many [class periods] are you required to attend in total?), METASPAM (Meta-cognition: assess credibility), ESCS (Index of economic, social and cultural status), HISEI (Index highest parental occupational status) for reading; COMPETE (Competitiveness), HISEI, METASPAM, ESCS, ICTRES, CPERWEEK for mathematics; ICTRES, CPERWEEK, PISADIFF (Perception of difficulty of the PISA test), METASPAM, COMPETE, JOYREAD (Joy/Like reading), ESCS, HISEI for science. On the other hand, it is seen that some of the variables have a direct effect on achievement in more than one field, even at different levels. In this respect, the variables whose area of influence and intersections are revealed according to the results of the analyses are presented in the Fig 2 in a more understandable way.

When the Fig 2 is analysed in detail, it is seen that ICTRES, CPERWEEK, ESCS, METASPAM and HISEI variables are determined as the most important variables for science, mathematics and reading sections, despite their different degrees of importance. On the other hand, another remarkable result is that CPERFORLAN variable is important for the reading section, PISADIFF and JOYREAD variables are important for the science section, and COMPETE variable is important for both maths and science sections. In the following, the average importance coefficients of the variables presented in Table 3 regarding the degree of importance in the related field are explicitly included.

**Table 3:** Coefficient table of determined variables (reading, maths and science).

Section	Variable	Mean Importance Coefficient	Coefficient estimates (mi)
**Reading**	**ICTRES**	3.787874	0.124000
	**CPERFORLAN**	6.047681	0.815000
	**CPERWEEK**	7.266618	0.844000
	**METASPAM**	3.666450	0.124000
	**ESCS**	4.005724	0.263000
	**HISEI**	5.982262	0.406000
**Science**	**ICTRES**	7.935320	0.484000
	**CPERWEEK**	7.708998	0.340000
	**PISADIFF**	3.658415	0.030000
	**METASPAM**	8.875383	0.550000
	**COMPETE**	3.629179	0.029000
	**JOYREAD**	3.867357	0.219000
	**ESCS**	3.675014	0.206000
	**HISEI**	4.506991	0.285000
**Math**	**COMPETE**	7.060205	0.628000
	**HISEI**	6.696192	0.412000
	**METASPAM**	5.710628	0.291000
	**ESCS**	5.517689	0.188000
	**ICTRES**	4.761140	0.174000
	**CPERWEEK**	4.434677	0.073000

When Table 3 is analysed in detail; 6 variables are determined as the most important variables for the Reading section. It is seen that the variable with the highest average importance coefficient is CPERWEEK (7.266618) and the variable with the lowest importance coefficient is METASPAM (3.666450). When the coefficient estimates obtained from the average importance coefficient are analysed, the most important variable belonging to the Reading section is CPERWEEK (.844). The variables with the lowest prediction coefficient among the most important variables were METASPAM (.124) and ICTRES (.124).

For the mathematics section, 6 variables were determined as the most important variables. It is seen that the variable with the highest average importance coefficient is COMPETE (7.060205) and the variable with the lowest average importance coefficient is CPERWEEK (4.434677). When the coefficient estimates obtained in this section are analysed, it is seen that the highest coefficient of estimation is COMPETE (.628) and the lowest coefficient of estimation is CPERWEEK (.073).

In the science department, 8 variables were determined as the most important variable. It is seen that the variable with the highest average importance coefficient is METASPAM (8.875383) and the variable with the lowest importance coefficient is COMPETE (3.629179). When the coefficient estimates obtained in this section are analysed, the variable with the highest coefficient of estimation is METASPAM (.550) and the variable with the lowest coefficient of estimation is COMPETE (.029).

### RQ2: Proposal for PISA score prediction models

The equations obtained from the coefficient estimates obtained through the model are presented in Model 1 for Reading, Model 2 for Maths and Model 3 for Science.


$$\mathrm{\backslash [}Model\;1(reading)=0,643+\;\sum_{i=1}^{6}\left[coefficient\;estimation\;of\;item\;mi\right]\mathrm{\backslash ]}$$

$$\mathrm{\backslash [}Model\;2(math)=0,630+\;\sum_{i=1}^{6}\left[coefficient\;estimation\;of\;item\;mi\right]\mathrm{\backslash ]}$$

$$\mathrm{\backslash [}Model\;3(science)=0,589+\;\sum_{i=1}^{8}\left[coefficient\;estimation\;of\;item\;mi\right]\mathrm{\backslash ]}$$

When the total coefficient values to be obtained over the models are analysed, it is predicted that a country with a score of greater than 3,219 from the model obtained from the Reading section can be successful. It is predicted that a country with a score of greater than 2.396 in Maths and greater than 2.732 in Science will be successful. Based on these results, it is predicted that countries can use the results obtained from the models as an early warning system. Thus, the answer to the second research question was obtained.

## Discussion and conclusions

This study was conducted using data from PISA 2018 to identify the main factors influencing student performance in high-performing countries among OECD countries and to develop a predictive model based on these variables. Thanks to the RFA applied, not only non-linear relationships but also the interactions and rank order of importance of the variables were analysed. In this respect, the RFA went beyond classical statistical methods and offered more contextual and explanatory analysis possibilities; it recognised patterns that were not visible with traditional models [22,23]. One of the main reasons why the RFA was preferred is that it allows the simultaneous modelling of a large number of independent variables in high-dimensional data sets. This algorithm offers a great advantage, especially in terms of calculating the relative importance of the variables and eliminating multicollinearity problems [25]. However, the RFA algorithm does not directly show causal relationships, but only classifies the predictive power between the variables. Therefore, caution is required when interpreting the results obtained. It is recommended to test possible cause-effect relationships with supporting analyses.

The results of the analysis show that the most important variables influencing students’ PISA success are the following: Access to information technology in all subjects, weekly hours of instruction in each subject, metacognitive awareness, socioeconomic and cultural status, and parents’ occupation. In addition, weekly instruction in a foreign language was found to be significant for reading, competition in maths and science, perception of test difficulty, awareness and students’ emotional engagement in reading.

When examining the variables that influence PISA success, the variable of access to information technology should be mentioned first, which was identified as the most important variable for all areas. Tools and applications based on information technologies have been found to provide students with quick access to relevant sources of information, thereby facilitating their engagement in active learning environments and accelerating their learning processes [11,32–34]. On the other hand, the level of awareness of the use of technological tools and the use of relevant tools for educational purposes are very important for students’ academic success. Among the variables that affect PISA success, economic, social and cultural status and parents’ occupation are usually mentioned together. Socioeconomic status, family cultural structure, parents’ occupation, and their attitudes toward education have been shown to directly influence students’ academic success in numerous studies [35–38]. As a study by [39] shows, Vietnam, despite being one of the poorest countries in the world, performed very well in maths and reading in the 2012, 2015 and 2018 PISA exams compared to many other countries with high economic power. In this regard, it can be said that the economic level or socio-cultural disadvantages of students can be partially overcome with a good coaching and mentoring system. On the other hand [40], have clearly shown in their studies the contribution of parental support to students’ success. There are also various studies in the literature that support the idea that parental support is directly related to the parenting skills of the parents in question [41].

When examining the variable of weekly instructional hours, which is one of the most important variables influencing PISA results, it is generally found that in all three subjects, the length of weekly instructional hours devoted to learning in the subject in question, i.e., the amount of time devoted to learning, has a direct impact on students’ academic performance [42]. state in their study that time management is a variable that should be considered as part of the implementation of a correct educational policy. Similarly, this also applies to the variable of weekly lessons in a foreign language, which was identified as an important variable based on the reading section. The amount and continuity of lessons play a crucial role in improving students’ grammar, vocabulary and reading strategies [43].

Among the main variables that influence PISA success, another variable that attracts attention is metacognition. Metacognition refers to a person’s ability to understand, control and regulate their own thought processes. The relationship between academic success and metacognition is related to students’ ability to manage learning processes more effectively. As [44] emphasise, students with metacognitive skills are more likely to be able to set learning goals, develop strategies, monitor their learning progress and make adjustments when necessary. As [45] suggests, the use of metacognitive processes increases students’ deep understanding, problem-solving skills and critical thinking. This helps them to be more successful in exams, projects and overall academic performance. Metacognition is often positively associated with academic success because it allows students to be more intentional about their learning processes. Metacognition can therefore have a positive impact on students’ academic success as it enables them to utilise learning strategies effectively.

The variable that has a direct impact on PISA success in maths is the variable competition. Competition refers to the rivalry between individuals or groups to achieve certain goals. This competition is often an incentive for better performance, success or the achievement of a specific goal. Maths lessons, where what is right or wrong can be clearly defined, provide a more suitable basis for performance comparisons, which encourages competition. However, in subjects such as psysical education, science and reading, competition is not so obvious as the subject is often based on subjective judgement and collaboration [46]. In these subjects, a variety of skills are often emphasised and the focus is on collaboration and understanding rather than competition.

Other unique variables that directly impact PISA success in science include two variables: emotional state related to the act of reading and perception of the difficulty of the PISA test. The enjoyment of reading supports students’ ability to understand and evaluate scientific texts. Positive reading motivation promotes deeper learning by increasing students’ interest in scientific topics. On the other hand, the perception of the difficulty of the test, i.e., the perception of the quality of the content of the texts read, influences the students’ attitude and their success in the exams. A high perception of difficulty creates stress and anxiety in students. This can lead to poor performance in science classes. Therefore, it is important that students are helped to develop a positive attitude towards the exams. However, the effects of these two variables can vary from student to student. As suggested by [47], students’ individual characteristics, learning styles and motivation levels interact with these factors. It is important for teachers to understand how students respond to these factors and support their individual learning needs in order to increase learning success in science courses.

### Strategic recommendations for practitioners

The results obtained provide concrete data that can be utilised to shape educational policy. In this context, improving digital accessibility should not be limited to the provision of hardware, but should also be supported by the development of digital pedagogical content for teachers and students. The integration of metacognitive skills into the curriculum, especially through self-directed learning strategies, will help students to manage their learning processes more consciously and effectively. It is important to increase the number of lessons only quantitatively and to focus this process on learning outcomes by enriching it with skilled content. The competitive environment should be constructively encouraged, especially in areas that require objective assessment, such as maths. However, supporting co-operative learning activities in subjects such as science and reading can lead to more effective outcomes. In addition, counselling services for economically disadvantaged students and support systems that involve the family should be strengthened, which will help to reduce inequalities in education [39].

### Limitations and future research

This study has some methodological limitations. First of all, the analysis covers only 37 OECD member countries. A direct generalisation of the results to countries outside the OECD is therefore only possible to a limited extent and should be interpreted taking contextual differences into account. Furthermore, the study was limited to data at student level; variables at teacher, school or system level were excluded from this model. This restricts the scope of the model and limits the impact analysis to a specific level. In future studies, the use of statistical models at multiple levels, the combination of different data sources and the preference for mixed methods supported by qualitative interviews will help to obtain results that are both generalisable and explanatory.

### General evaluation

This study has made the multidimensional structures behind the PISA success more visible by utilising the powerful classification and determination of the degree of importance of variables by the RFA, providing a remarkable framework for evidence-based policy making in education. The flexibility that RFA offers in explaining complex interactions that are often ignored by traditional methods is a significant advantage, especially in multivariate systems such as education [20,22]. The insights gained not only provide directional information about the factors that influence student success, but also about which components of the education system to focus on.

Indeed, recent OECD reports [48,49] clearly emphasise the influence of students’ access to digital resources, levels of metacognitive awareness and time spent learning on long-term success. The results of this research also support and strengthen these findings through a machine learning-based approach to analysis. RFA can therefore be evaluated not only as a classification tool, but also as a strategic method that can contribute to the development of more equitable,student-centred and effective educational policies.

In future studies, the application of similar methods in different groups of countries, educational systems or disciplines will both prepare the ground for comparative analyses and allow stronger conclusions to be drawn about the generalisability of this model.

## Supporting information

S1 Data Dataset containing the variables influencing PISA scores. (XLSX)
